# Temporary Resection of the Cranial Bones as a Substitute for Trephining

**Published:** 1890-04

**Authors:** 


### Selections and Abstracts.

### TEMPORARY RESECTION OF THE CRANIAL BONES AS A SUBSTITUTE FOR TREPHINING.

The operation of trephining, which is now-a-days so frequently performed, has the disadvantage of leaving the patient with an opening in the skull, which, although closed in most instances by the formation of a firm mass of cicatricial tissue, frequently necessitates the employment of an artificial protector. To overcome this difficulty surgeons have practiced with more or less success transplantation of the excised disk of bone, or of bone taken from animals. For some time past Dr. W. Wagner, of Germany (*Centralblatt fur Chirurgie*, Nov. 23, 1889), has experimented on the cadaver with the following method, which is easy of execution and possesses the great advantage of not completely detaching the resected bone from the surrounding parts, and thus insures its healing into the defect.

An incision, shaped like the Greek letter omega, is made through the scalp down to the periosteum. The latter is then divided by a similar cut running parallel and somewhat within the first, and the bone is chiselled in the line of the periosteal incision. The intervening bridge of bone at the base of the omega is divided subcutaneously with small chisels, care being exercised not to inflict injury to the surrounding soft parts. The exsected plate of bone is lifted up with small elevators and retracted with its coverings, and remains attached to the surrounding integuments by a pedicle 3 cm. in width, which is situated at the base of the omega.

After the performance of the operation the retracted flap is returned to its original position. The pericranial flap is carefully sutured, and drainage tubes are inserted in the angles at the base of the omega. If the edges of the internal lamella of the excised bone, which usually remain attached to the margins of the defect, are not chiselled away, the bone will perfectly fit in the opening, and this will prevent any possible depression of the fragment.

The author has only had an oportunity to employ this procedure in a single case, and found it as easy of execution on the living as on the dead body. The patient had sustained a fracture at the base of the skull, with rupture of the left middle meningeal artery, and the operation was undertaken to relieve the existing pressure symptoms from the clot. In this respect it fulfilled its object, but owing to the presenee of severe complicating brain injury, death ensued at the end of twenty-four hours. It is a noteworthy fact, however, that at the autopsy the excised flap of bone and soft parts were found in a perfectly healthy condition.—*International Journal of Surgery.*

